# Cardiovascular magnetic resonance myocardial feature tracking using a non-rigid, elastic image registration algorithm: assessment of variability in a real-life clinical setting

**DOI:** 10.1186/s12968-017-0333-y

**Published:** 2017-02-17

**Authors:** Pedro Morais, Alberto Marchi, Julie A. Bogaert, Tom Dresselaers, Brecht Heyde, Jan D’hooge, Jan Bogaert

**Affiliations:** 1 Lab on Cardiovascular Imaging & Dynamics, Department of Cardiovascular Sciences, KULeuven - University of Leuven, Herestraat 49, Leuven, Belgium; 2 ICVS/3B’s - PT Government Associate Laboratory, Braga/Guimarães, Portugal; 3 Instituto de Engenharia Mecânica e Gestão Industrial, Faculdade de Engenharia, Universidade do Porto, Porto, Portugal; 4 Department of Imaging and Pathology, KU Leuven – University of Leuven, Herestraat 49, Leuven, Belgium

## Abstract

**Background:**

Cardiovascular magnetic resonance myocardial feature tracking (CMR-FT) is a promising technique for quantification of myocardial strain from steady-state free precession (SSFP) cine images. We sought to determine the variability of CMR-FT using a non-rigid elastic registration algorithm recently available in a commercial software package (Segment, Medviso) in a real-life clinical setting.

**Methods:**

Firstly, we studied the variability in a healthy volunteer who underwent 10 CMR studies over five consecutive days. Secondly, 10 patients were selected from our CMR database yielding normal findings (*normal group*). Finally, we prospectively studied 10 patients with known or suspected myocardial pathology referred for further investigation to CMR (*patient group*). In the patient group a second study was performed respecting an interval of 30 min between studies. All studies were manually segmented at the end-diastolic phase by three observers. In all subjects left ventricular (LV) circumferential and radial strain were calculated in the short-axis direction (Ecc_SAX_ and Err_SAX,_ respectively) and longitudinal strain in the long-axis direction (Ell_LAX_). The level of CMR experience of the observers was 2 weeks, 6 months and >20 years.

**Results:**

Mean contouring time was 7 ± 1 min, mean FT calculation time 13 ± 2 min. Intra- and inter-observer variability was good to excellent with an coefficient of reproducibility (CR) ranging 1.6% to 11.5%, and 1.7% to 16.0%, respectively and an intraclass correlation coefficient (ICC) ranging 0.89 to 1.00 and 0.74 to 0.99, respectively. Variability considerably increased in the test-retest setting with a CR ranging 4.2% to 29.1% and an ICC ranging 0.66 to 0.95 in the patient group. Variability was not influenced by level of expertise of the observers. Neither did the presence of myocardial pathology at CMR negatively impact variability. However, compared to global myocardial strain, segmental myocardial strain variability increased with a factor 2–3, in particular for the basal and apical short-axis slices.

**Conclusions:**

CMR-FT using non-rigid, elastic registration is a reproducible approach for strain analysis in patients routinely scheduled for CMR, and is not influenced by the level of training. However, further improvement is needed to reliably depict small variations in segmental myocardial strain.

**Electronic supplementary material:**

The online version of this article (doi:10.1186/s12968-017-0333-y) contains supplementary material, which is available to authorized users.

## Background

Evaluation of cardiac performance is crucial in daily clinical practice to evaluate disease severity, to assess therapeutic interventions and to predict outcome [1]. The lack of easy-to-determine indices of myocardial contractility imposed the cardiology community to consider ejection fraction (EF) the reference standard of ventricular systolic function despite its inability to evaluate regional contractility and its poor sensitivity to detect subtle alterations in ventricular function [2]. Myocardial strain, defined as the measurement of the fractional change of myocardial dimension in a specific direction, appears a superior parameter to assess both global and regional myocardial function. Furthermore, it has been shown to be an early marker of systolic dysfunction and to precede decline in ejection function [3].

Over the years, cardiovascular magnetic resonance (CMR) has emerged as the reference standard for the evaluation of ventricular morphology and function, including myocardial strain [4–6]. CMR tagging, introduced about 25 years ago, enables noninvasive quantification of myocardial deformation and strain calculation [7, 8], but due to the need of dedicated tagging sequences and time-consuming analysis has not gained widespread acceptance in the clinical arena [9, 10].

Feature tracking (FT) has recently been introduced for analysis and quantification of myocardial strain based on cine steady-state free-precession (SSFP) images, which are part of a standard study CMR protocol [11–13]. Although the feature tracking algorithm was originally developed for echocardiographic imaging studies, it has recently been introduced for analysis and quantification of myocardial strain based on cine steady-state free-precession (SSFP) images [11–13]. Important advantages are that this technique does not require additional time consuming sequences (as for CMR tagging) but can be applied to standard cine SSFP exams thereby leaving the potential to retrospectively analyze strain in patients where no specific strain image acquisition sequence was performed. Moreover, it has the advantage that the analyses can be performed in a retrospective manner. The CMR-FT algorithm is based on optical flow technology as commercially introduced by TomTec (Unterschleissheim, Germany). More recently, Circle (cvi, Calgary, Canada) has provided similar CMR-FT software. These algorithms use the left ventricular (LV) boundaries to estimate myocardial strain and strain rates in short (SA) and long-axis (LA) direction. In the present study, we have used a non-rigid, elastic image registration algorithm available from Segment (Medviso, Lund, Sweden) [14, 15]. Although reproducibility values for CMR-FT have been published, all used the optical flow algorithm [16–22]. Accordingly, the aim of the present study was to investigate not only intra- and inter-observer variability but also inter-study reproducibility of CMR-FT in a setting reflecting the real clinical practice. Hereto, both subjects with and without pathologic findings at CMR were included and observers with a different level of expertise were involved.

## Methods

### Study population

*Part 1/*One healthy male volunteer (age 43) underwent 10 consecutive CMR studies over a period of 5 days, i.e., two CMR studies per day. Although per day, the CMR studies were subsequently performed, for the second CMR study the volunteer was re-installed and a new exam was started obtaining new localizers and a new determination of cardiac axes was performed. No Gadolinium-based contrast agent was administered.

*Part 2*/From the UZ Leuven patient CMR database (University Hospitals Leuven, Belgium) we randomly selected 10 patients (7 male) with suspected cardiomyopathy but normal CMR findings (*normal group*). Mean age 37 ± 11 years; mean LVEF 57 ± 5%. Each patient underwent a complete CMR study with intravenous administration of Gadolinium-based contrast agent (Gadovist, Bayer).

*Part 3*/Ten patients (6 male) with known or suspected ischemic or non-ischemic myocardial disease were prospectively enrolled (*patient group*). Each patient was scanned twice with an interval of 30 min between the two CMR studies after re-installing the patient on the scanner. Gadolinium-based contrast agent (Gadovist, Bayer) was injected once, i.e., in the first exam.

Renal function (eGFR) was checked in all subjects prior to contrast agent administration. Exclusion criteria were standard contra-indications to MRI.

### Cardiovascular magnetic resonance acquisition

All studies were performed on a 1.5 T unit (Ingenia; Philips Healthcare, the Netherlands) by using commercially available CMR software, electrocardiographic triggering, and a cardiac-dedicated phase-array coil. For the assessment of LV dimensions and function bSSFP breath-hold cine images were acquired in the following orientations: vertical long axis, horizontal long axis and short axis. Standard parameters were repetition time/echo time 3.6/1.8 ms; sense factor 2, flip angle, 60°; section thickness, 8 mm; matrix, 160 × 256; field of view, 300 mm; pixel size, 1.6 × 1.6 mm; and number of phases, 30, phase percentage, 67%. The vertical long axis was determined on the transverse images by positioning an image plane connecting the middle of the mitral valve with the LV apex. On the vertical long axis, the same anatomical landmark points were used to define the horizontal long axis plane. The cardiac short axis was defined on the horizontal long axis using an image plane perpendicular to the interventricular septum. Care was taken to position the most basal short axis slice at end-diastole exactly through the mitral valve ring. The set of short axis images encompassed the left ventricle entirely. Between slices a gap of 2 mm was used. Per breath hold two short axis cine CMR were acquired. For the Late Gadolinium Enhancement (LGE) studies, a dose of 0.15 mL of gadobutrol (Gadovist, Bayer) per kilogram of body weight, was administered.

### Left ventricular volumes and function

The image analysis was performed offline by a single observer with more than 20 years of experience in CMR. LV volumes and function were measured using manual planimetry of the endocardial and epicardial borders from the short-axis set, in accordance with approved protocols using a commercially available software package (ViewForum; Philips Medical Systems, Best, the Netherlands) (see online data material).

### Feature tracking

**Fig. 1:**
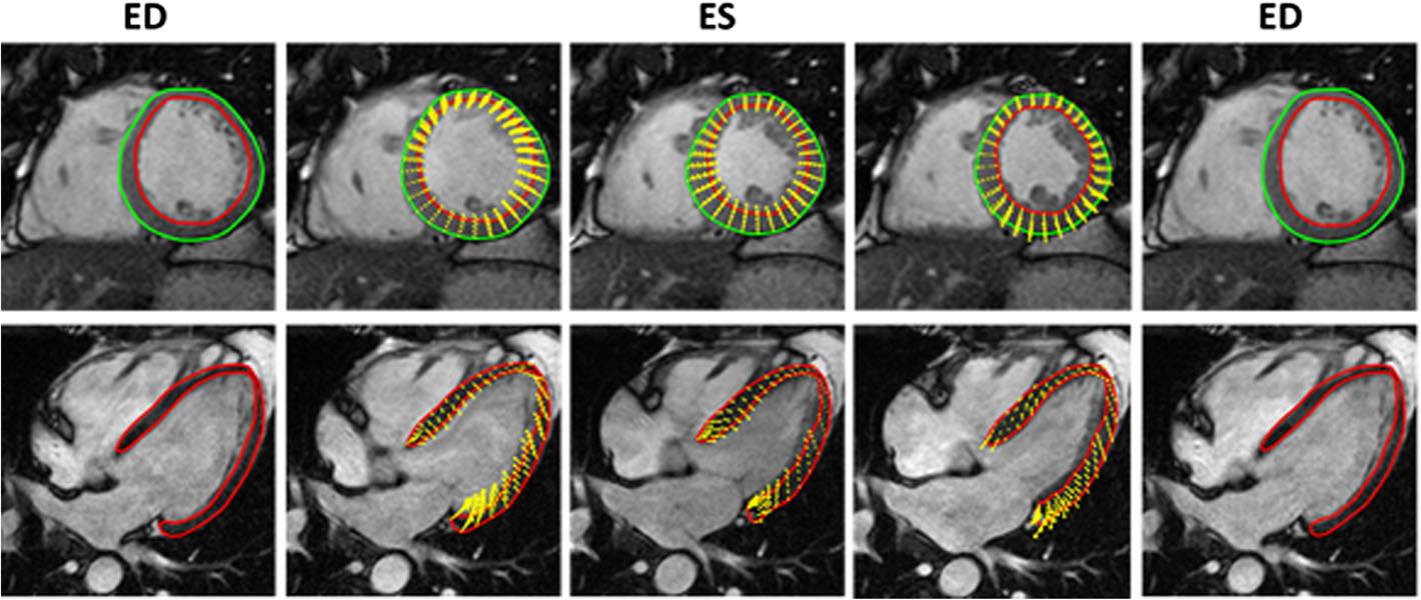
Feature tracking in a patient belonging to the normal group in short-axis (*upper panels*) and horizontal long-axis (*lower panels*). Manual delineation of the endo- and epicardial contour at the end-diastole (*left panels*). Next, the software automatically deforms these contours using a dense motion field (*yellow arrow*) estimated between consecutive frames

Myocardial strain analysis was performed using Segment Medviso software. This software estimates myocardial strain curves by computing inter-frame deformation fields using a tracking strategy based on non-rigid image registration [14, 15]. Specifically, the deformation field is modeled using a B-spline tensor product transform and is found by deforming one image to the next image in the cine sequence. As such, a dense map that describes the difference between consecutive images for all image positions is obtained. The deformation field is computed through an iterative process guided by an intensity-based similarity metric combined with a regularization term, which enforces spatial smoothness of the recovered deformation field. Note that, instead of myocardial boundaries tracking only, the proposed method use the entire image content (i.e. blood pool, entire myocardium) during the optimization process. Furthermore, in order to increase the robustness of the technique to bad image quality, noisy image and image artifacts, a temporal coherence strategy was proposed. Indeed, the tracking methodology was reformulated to take the temporal information of the entire cardiac sequence into account at once. As a result, a strategy that efficiently penalizes non-smooth deformations while keeping a high enough flexibility to extract the relevant physiological deformation was obtained. Fig. 1 shows an example of the tracking result in a normal patient. Circumferential and radial LV myocardial Lagrangian strain were evaluated on short-axis cine SSFP CMR. Longitudinal Lagrangian strain was derived from both horizontal and vertical long-axis cine SSFP CMR. First, both endo- and epicardial contours were manually drawn at end diastole in the long and short-axis by three readers blinded to patients details and to the results of the other readers. Note that, both experts used the same strategy to perform the delineation. Furthermore, it should be noted that the initial contour defines the relevant components of the dense deformation field for computation of the myocardial strain. Contours were propagated automatically by the software throughout the cardiac cycle generating myocardial strain and strain rate curves. No drift correction was performed. Note that, in case the tracking was suboptimal, the original contour (usually the end-diastolic image) could be manually adjusted and re-propagated without the need to rerun the tracking algorithm completely. Subsequently, a reference axis was manually positioned on the mid-septum in order to establish left ventricle segmentation using the 16-segment approach as defined by the American Heart Association [23]. The level of CMR experience varied between the three readers, *expert observer*, i.e., >20 years of CMR experience (SCMR level III) and skilled observer i.e., 6 months of CMR experience (EACVI CMR level 2) and a *beginner* i.e., two weeks of training in CMR analysis (basic knowledge of cardiac anatomy, cardiac imaging planes, and training in cardiac image contouring). To assess the intra-observer variability, the skilled reader re-assessed the CMR studies one week after the first reading, unaware of the initial study results.

### Statistics

Statistical analysis was performed using IBM SPSS Statistics version 20 and Microsoft Excel 2013. All variables with normal distribution are presented as mean ± standard deviation. In the short-axis direction, only slices that were contoured by both observers, or during both readings were considered for calculation of the variability. Next, the position of the papillary muscles was used to identify the basal, mid-ventricular and apical level.

In order to assess inter-study, inter- and intra-observer variability, the intraclass correlation coefficient (ICC), Bland-Altman analysis, coefficient of repeatability (CR) and the coefficient of variation (CV) were computed using the strain value at end-systole (ES). Note that ES timing was defined for each dataset by one expert. The analysis was performed using segmental and global strain values. Segmental values were defined using the AHA 16 segments model [23], while global strain was computed as the mean value of all segments in each level. Note that for the radial and circumferential strain computation only the short-axis slices delineated by all the readers were considered. Long-axis strain was computed using horizontal and vertical long axis acquisitions. For the Bland-Altman analysis, both bias (mean differences) and limits of agreement (LOAs, 1.96 times the standard deviation of the differences) were computed. The CV was defined as the standard deviation of the differences (between multiple readings of the same case) divided by the mean value of all samples [22]. CR is computed as 2.77 multiplied by the standard deviation of the differences between readings, as described in [24]. Regarding the healthy volunteer experiment (10 acquisitions), the CV, bias and LOA was computed using the difference between each acquisition against the first one. A two-tailed paired *t*-test against zero was used to check for statistically significant differences (p < 0.05) of the observer biases. Moreover, the inter-study, inter- and intra-observer variability was compared through two-tailed F-test’s (p < 0.05). The D’Agostino-Pearson test was used to determine normality of the data. The global strain values in both normal and patient group were compared with LV ejection fraction, using Pearson correlation coefficient.

## Results

Demographics of the healthy volunteer, and the subjects in the normal and patient group are summarized in the online addenda (Additional file 1). CMR in the patient group, consisting of a mix of patients with acute and chronic ischemic and non-ischemic myocardial disease, yielded normal findings in two patients (i.e., referral for ventricular extrasystoles and familial history of hypertrophic cardiomyopathy). One long-axis cine study in the patient group was because of motion artefacts not useful for analysis. Time for contouring a complete dataset (horizontal long axis - HLA, vertical long axis – VLA, cine and complete SA cine stack) was 7 ± 1 min, and did not show differences between observers (p = 0.56). Myocardial strain calculation time (Dell XPS 8500 Desktop, Intel i7, 16Gb) for this dataset was 13 ± 2 min.

**Fig. 2:**
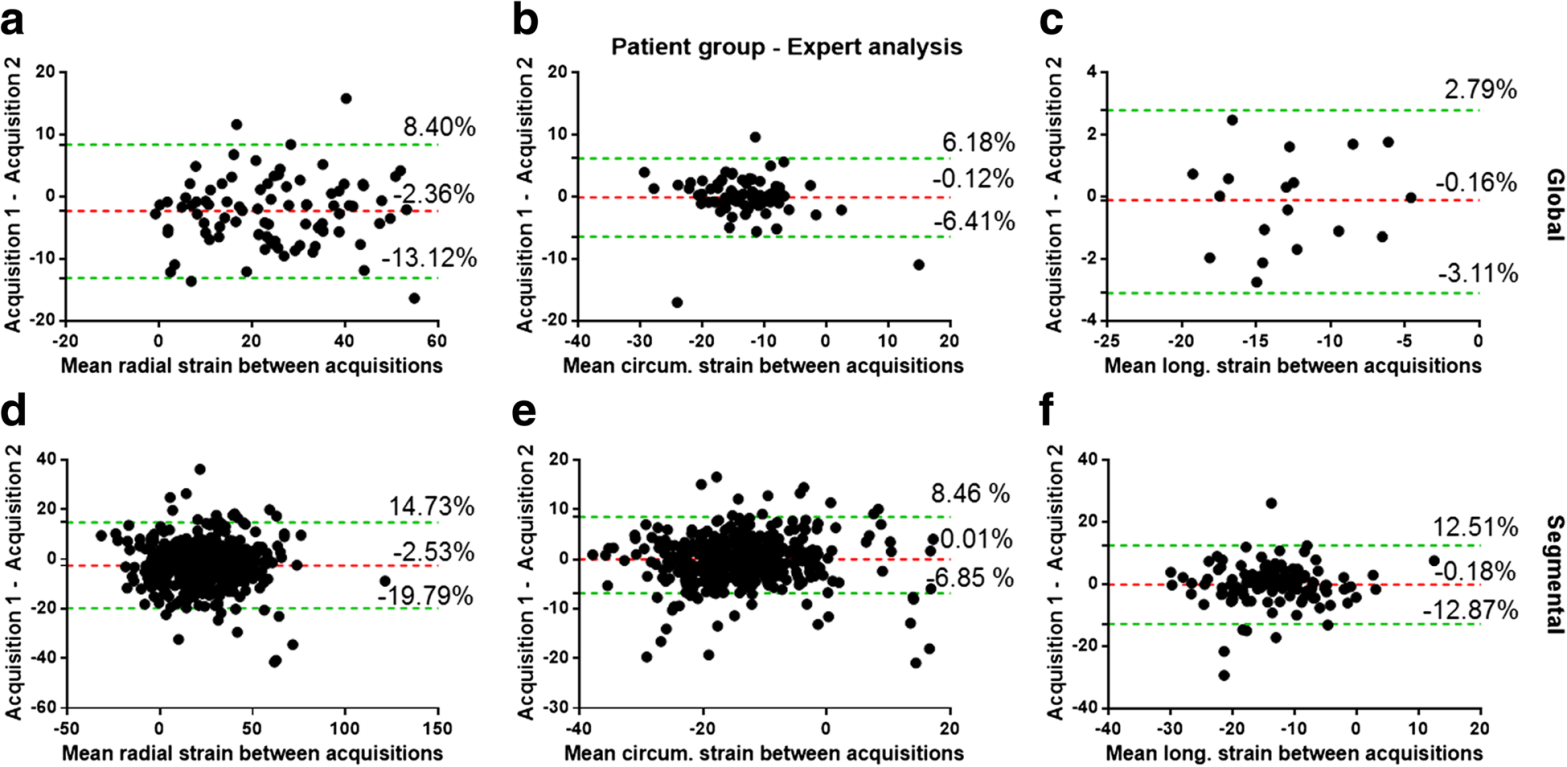
Reproducibility study using the multiple acquisitions of the patient group. Bland-Altman plots for global and segmental ES (**a**, **d**) radial, (**b**, **e**) circumferential and (**c**, **f**) longitudinal strain obtained. Dashed lines represent bias (*red*) and 95% limits of agreement (**b**)

**Fig. 3:**
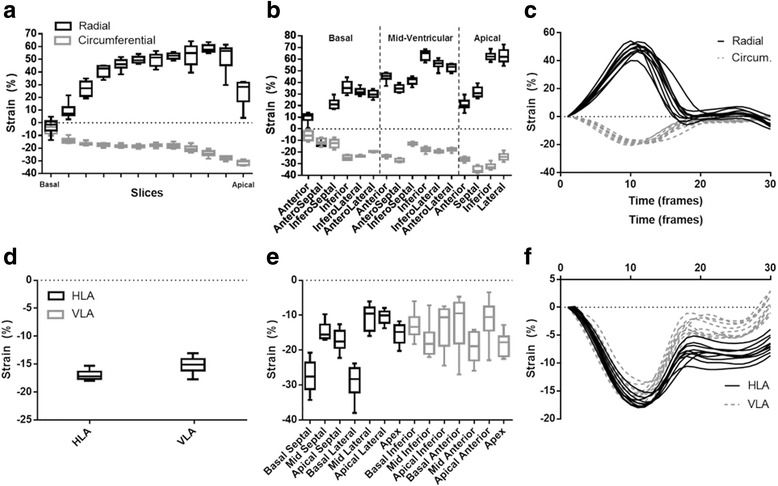
Mean strain and variability per slice level and per segment in the healthy volunteer (10 acquisitions). **a**, **d** represents the global strain components and **b**, **e** the segmental strain. **c** shows the obtained global radial and circumferential strain curves in a mid-ventricular slice for all the 10 acquisitions. **f** presents the obtained strain curve for the global longitudinal strain

Mean strain values are shown in Table 1. With regard to variability and myocardial strain, a/the variability was systemically the largest for radial strain, b/variability systemically increased from global to segmental level, and c/variability increased from midventricular to the basal and apical LV level (see Tables 2, 3, 4 and 5 and Fig. 2). For instance, CR for global radial and circumferential strain in the healthy volunteer were 3.6% and 2.6%, 8.8% and 3.7%, and 12.0% and 3.7% for intra-observer, inter-observer and inter-study variability, respectively. Similar values were found in the normal and patient group. Compared to global strain variability, segmental strain variability almost invariably showed two-to threefold higher CR values, e.g. CR for intra-observer variability in the patient group ranged 1.6% to 2.7% for global strains versus 4.7% to 10.1% for regional strain estimation (Table 2). As shown in Fig. 3 and in Table 6, the lowest standard-deviations for variability for radial and circumferential strain were in the midventricular part of the left ventricle but considerably enlarged towards the LV base and LV apex. For segmental longitudinal strain variability, a less consistent pattern was found (Table 7 and Fig. 3).

**Table 1:** Mean (%) and standard deviation (STD, %) myocardial strain values (at end-systole) in the healthy volunteer, the normal and patient group

	Mean	STD
Healthy volunteer		
ErrSAX	38.6	5.0
EccSAX	−19.0	1.6
EllLAX	−16.1	1.5
Normal group		
ErrSAX	25.8	4.1
EccSAX	−15.8	2.8
EllLAX	−16.6	2.5
Patient group		
ErrSAX	23.9	10.4
EccSAX	−13.4	4.3
EllLAX	−12.9	4.4

**Table 2:** Intra-observer variability (by the skilled observer)

	Intra-observer		#	Bias	LOA	CV	CR	ICC (95% CI)
Healthy Volunteer	Global	ErrSAX	120	−0.33*	[−2.9;2.2]	4.7	±3.6	0.98 (0.98–0.98)
		EccSAX	120	−0.13	[−2.0;1.7]	5.0	±2.6	0.99 (0.98–0.99)
		EllLAX	20	0.06	[−1.3;1.4]	4.4	±1.9	0.89 (0.73–0.95)
	Segm.	ErrSAX	660	−0.25	[−8.4;7.9]	12.3	±11.5	0.98 (0.98–0.99)
		EccSAX	660	−0.12	[−5.8;5.6]	15.1	±8.1	0.96 (0.96–0.97)
		EllLAX	140	0.14	[−5.9;6.2]	19.4	±8.6	0.89 (0.85–0.92)
Normal Group	Global	ErrSAX	87	−0.22*	[−2.0;1.6]^§^	3.7	±2.6	1.00 (1.0–1.0)
		EccSAX	87	0.08	[−1.1;1.3]	3.7	±1.7	0.99 (0.99–1.0)
		EllLAX	20	0.07	[−1.4;1.5]	5.2	±2.0	0.95 (0.89–0.98)
	Segm.	ErrSAX	470	−0.18	[−7.7;7.4]^§^	15.3	±10.7	0.98 (0.97–0.98)
		EccSAX	470	0.12	[−4.2;4.5]	13.3	±6.2	0.97 (0.96–0.97)
		EllLAX	140	0.01	[−5.1;5.1]	15.0	±7.2	0.95 (0.93–0.97)
Patient Group	Global	ErrSAX	86	−0.23*	[−2.1;1.7]^§^	4.1	±2.7	1.00 (1.0–1.0)
		EccSAX	86	−0.16*	[−1.3;1.0]	4.7	±1.6	0.99 (0.99–1.0)
		EllLAX	18	0.34	[−1.2;1.9]	6.3	±2.2	0.98 (0.95–1.0)
	Segm.	ErrSAX	470	−0.20	[−5.7;5.3]^§^	11.7	±7.8	0.99 (0.99–0.99)
		EccSAX	470	−0.12	[−3.5;3.2]	12.9	±4.7	0.98 (0.98–0.98)
		EllLAX	126	0.24	[−6.9;7.4]	28.4	±10.1	0.90 (0.86–0.93)

# - Sample Size; *LOA* Limits of agreement; *CV* Coefficient of variation (%); *CR* Coefficient of repeatability; *ICC* Intraclass correlation coefficient; *CI* Coefficient Interval

*p < 0.05, two tailed paired *t*-test against zero

^§^ *p* < 0.05, F-test between patient group and normal group for each strain component

**Table 3:** Inter-observer variability in three observers with different level of expertise

	Inter-observer		#	Expert versus Skilled Observer					Expert versus Beginner				
				Bias	LOA	CV	CR	ICC (95% CI)	Bias	LOA	CV	CR	ICC (95% CI)
Healthy Volunteer	Global	ErrSAX	120	0.01	[−3.3;3.4]	4.3	±4.8	0.97 (0.97–0.98)	−0.61*	[−6.8;5.6]	8.3	±8.8	0.99 (0.98–0.99)
		EccSAX	120	0.46*	[−1.5;2.5]	5.4	±2.9	0.98 (0.98–0.98)	−0.34*	[−2.9;2.3]	*6.9*	±3.7	0.98 (0.97–0.99)
		EllLAX	20	0.50*	[−0.7;1.7]^£^	4.0	±1.7	0.85 (0.46–0.95)	0.70*	[−2.4;3.8]^£^	5.3	±2.2	0.74 (0.45–0.89)
	Segm.	ErrSAX	660	0.01	[−8.8;8.8]^£^	16.4	±12.4	0.96 (0.96–0.96)	−0.43	[−11.8;10.9]^£^	22.9	±16.0	0.95 (0.94–0.95)
		EccSAX	660	0.55*	[−5.6;6.7]^£^	*17.1*	±8.7	0.95 (0.94–0.96)	−0.22	[−10.2;9.8]^£^	*22.7*	±14.2	0.92 (0.91–0.93)
		EllLAX	140	0.60*	[−5.3;6.5]^£^	18.7	±8.3	0.90 (0.86–0.93)	0.86*	[−8.4;10.1]^£^	19.6	±13.1	0.80 (0.76–0.90)
Normal Group	Global	ErrSAX	87	0.57*	[−2.7;3.8]	5.7	±4.6	0.97 (0.97–0.97)	0.19 *	[−3.4;3.8]	7.3	±5.1	0.97 (0.96–0.97)
		EccSAX	87	0.56*	[−1.3;2.4]	5.2	±2.6	0.99 (0.98–0.99)	−0.16	[−1.8;1.4]	5.0	±2.2	0.99 (0.98–0.99)
		EllLAX	20	−0.03	[−1.5;1.5]	4.4	±2.2	0.89 (0.73–0.95)	0.29*	[−1.4;2.0]	5.2	±2.4	0.91 (0.87–0.96)
	Segm.	ErrSAX	470	0.57*	[−8.8;9.9]^§£^	21.3	±13.2	0.92 (0.89–0.95)	0.14	[−10.6;10.9]^§£^	21.8	±15.2	0.92 (0.91–0.94)
		EccSAX	470	0.55*	[−4.4;5.5]	15.0	±7.0	0.96 (0.96–0.97)	−0.18	[−5.5;5.1]^£^	17.3	±7.5	0.94 (0.93–0.95)
		EllLAX	140	−0.17	[−4.5;4.2]	19.4	±6.2	0.89 (0.85–0.92)	0.18	[−5.2;5.5]^§^	19.7	±7.5	0.90 (0.86–0.92)
Patient Group	Global	ErrSAX	86	0.26	[−3.2;3.7]	8.6	±4.9	0.97 (0.97–0.99)	0.09	[−2.8;3.0]	6.7	±4.1	0.99 (0.98–0.99)
		EccSAX	86	0.51*	[−1.0;2.0]	*6.0*	±2.2	0.99 (0.96–0.99)	0.13	[−1.5;1.7]	6.2	±2.2	0.98 (0.97–0.99)
		EllLAX	18	0.71*	[−1.2;2.7]	7.9	±3.7	0.97 (0.85–0.99)	−0.15	[−2.5;2.2]	7.9	±3.3	0.97 (0.88–0.99)
	Segm.	ErrSAX	470	0.26	[−8.2;8.7]^§£^	25.2	±11.9	0.93 (0.90–0.95)	0.13	[−8.6;8.9]^§£^	23.9	±12.4	0.92 (0.88-0.95)
		EccSAX	470	0.63*	[−4.5;5.8]^£^	*19.4*	±7.3	0.95 (0.94–0.96)	0.14	[−4.9;5.2]^£^	*21.0*	±7.2	0.95 (0.93-0.96)
		EllLAX	126	0.65*	[−5.7;7.0]	24.8	±9.0	0.92 (0.88–0.94)	0.44*	[−6.1;7.0]^§^	25.7	±9.3	0.90 (0.88–0.94)

# - Sample Size, *LOA* Limits of agreement; *CV* Coefficient of variation (%); *CR* Coefficient of repeatability; *ICC* Intraclass correlation coefficient; *CI* Coefficient Interval

*p < 0.05, two tailed paired *t*-test against zero

^§^ *p* < 0.05, two tailed F-test between patient group and normal group for each strain component

^£^ *p* < 0.05, two tailed F-test between the results obtained by the different observers (expert vs skilled observer, and expert versus beginner)

**Table 4:** Inter-study variability in the healthy volunteer

Inter-study			#	Bias	LOA	CV	CR	ICC (95% CI)
Expert	Global	ErrSAX	120	1.34*	[−7.1;9.8]^£^	22.0	±12.0	0.87 (0.74–0.95)
		EccSAX	120	0.26	[−2.3;2.9]	13.5	±3.7	0.90 (0.79–0.97)
		EllLAX	20	−1.79*	[−3.3;-0.3]^£^	9.6	±2.1	0.52 (0.10–1.00)
	Segm.	ErrSAX	660	−0.23	[−11.8;11.4]^$£^	30.7	±16.4	0.86 (0.80–0.91)
		EccSAX	660	1.02*	[−4.3;6.3]	26.8	±7.5	0.84 (0.78–0.89)
		EllLAX	140	0.66*	[4.8;6.1]^£^	33.4	±7.7	0.60 (0.41–0.81)
Skilled Observer	Global	ErrSAX	120	1.53*	[−7.2;10.3]	22.8	±12.4	0.86 (0.72–0.95)
		EccSAX	120	0.08	[−2.8;3.0]	15.5	±4.1	0.88 (0.77–0.96)
		EllLAX	20	−1.56*	[−3.2;0.1]	10.4	±2.3	0.50 (0.08–1.00)
	Segm.	ErrSAX	660	−0.29	[−11.0;10.4]^$^	28.3	±15.1	0.87 (0.81–0.91)
		EccSAX	660	0.95*	[−4.0;5.9]	25.7	±7.0	0.87 (0.83–0.91)
		EllLAX	140	0.86*	[−4.2;6.0]	32.2	±7.3	0.60 (0.40–0.80)
Beginner	Global	ErrSAX	120	0.83	[−8.5;10.1]^£^	24.6	±13.1	0.84 (0.69–0.94)
		EccSAX	120	0.13	[−3.0;3.3]	16.0	±4.5	0.88 (0.76–0.96)
		EllLAX	20	−2.06*	[−4.2;0.1]^£^	13.7	±3.1	0.40 (0.03–1.00)
	Segm.	ErrSAX	660	0.01	[−12.8;12.8]^£^	34.3	±18.1	0.83 (0.77–0.88)
		EccSAX	660	0.90*	[−5.0;6.8]	29.3	±8.3	0.83 (0.77–0.89)
		EllLAX	140	1.42*	[−5.7;8.5]^£^	45.6	±10.0	0.51 (0.36–0.78)

# - Sample Size; *LOA* Limits of agreement; *CV* Coefficient of Variance (%); *CR* Coefficient of repeatability; *ICC* Intraclass correlation coefficient; *CI* Coefficient Interval

*p < 0.05, two tailed paired *t*-test against zero

^$^ *p* < 0.05, two tailed F-test between Expert versus Skilled observer variability

^£^ *p* < 0.05, two tailed F-test between Expert versus Beginner variability

**Table 5:** Inter-study variability in the patient group

Inter-study			#	Bias	LOA	CV	CR	ICC (95% CI)
Expert	Global	ErrSAX	86	−2.25*	[−13.7;9.2]	23.7	±16.2	0.91 (0.85–0.95)
		EccSAX	86	−0.31	[−7.0;6.3]	27.1	±9.3	0.83 (0.75–0.89)
		EllLAX	18	0.04	[−2.9;3.0]^£^	12.3	±4.2	0.95 (0.87–0.98)
	Segm.	ErrSAX	470	−2.33*	[−21.7;17.0]^$£^	39.5	±27.3	0.86 (0.83–0.89)
		EccSAX	470	−0.16	[−10.7;10.3]^£^	40.7	±14.8	0.80 (0.76–0.83)
		EllLAX	126	−0.14	[−10.5;10.2]^$£^	41.7	±14.6	0.79 (0.71–0.85)
Skilled Observer	Global	ErrSAX	86	−2.36*	[−13.1;8.4]	22.5	±15.2	0.92 (0.86–0.95)
		EccSAX	86	−0.12	[−6.4;6.2]	24.9	±8.9	0.88 (0.82–0.92)
		EllLAX	18	−0.16*	[−3.1;2.8]	11.7	±4.2	0.94 (0.86–0.98)
	Segm.	ErrSAX	470	−2.53*	[−19.8;14.7]^$^	35.6	±24.4	0.88 (0.85–0.91)
		EccSAX	470	0.01	[−6.9;8.5]	33.4	±12.0	0.86 (0.83–0.88)
		EllLAX	126	−0.18	[−12.9;12.5]^$^	48.7	±17.9	0.66 (0.54–0.74)
Beginner	Global	ErrSAX	86	−2.41*	[−14.0;9.2]	24.1	±16.4	0.91 (0.84–0.94)
		EccSAX	86	−0.69	[−6.1;6.6]	26.3	±10.3	0.84 (0.76–0.89)
		EllLAX	18	−0.49	[−4.4;3.4]^£^	15.8	±5.5	0.90 (0.74–0.96)
	Segm.	ErrSAX	470	−2.47*	[−23.1;18.1]^£^	37.4	±29.1	0.86 (0.83–0.88)
		EccSAX	470	−0.65	[−12.0;10.7]^£^	43.0	±16.0	0.71 (0.67–0.74)
		EllLAX	126	−0.35	[−12.4;11.7]^£^	48.2	±17.0	0.77 (0.65–0.85)

# - Sample Size, *LOA* Limits of agreement; *CV* Coefficient of variation (#); *CR* Coefficient of repeatability; *ICC* Intraclass correlation coefficient

*p < 0.05, two tailed paired *t*-test against zero

^$^ *p* < 0.05, two tailed F-test between Expert versus Skilled observer variability

^£^ *p* < 0.05, two tailed F-test between Expert versus Beginner variability

**Table 6:** Radial and circumferential strain values obtained for the normal group

Segment	Radial			Circumferential		
	Basal	Mid-ventricular	Apical	Basal	Mid-ventricular	Apical
Anterior	26.0 ± 11.34	31.8 ± 8.4	26.2 ± 16.7	−12.4 ± 5.7	−15.6 ± 3.5	−18.0 ± 6.1
Anteroseptal	3.1 ± 17.2	22.1 ± 7.4	13.7 ± 14.7	−16.1 ± 10.5	−22.0 ± 3.7	−26.1 ± 6.0
InferoSeptal	11.7 ± 10.1	24.6 ± 8.5		−10.5 ± 5.9	−12.5 ± 5.6	
Inferior	29.3 ± 13.5	34.3 ± 12.7	34.6 ± 15.3	−16.2 ± 5.7	−15.2 ± 4.2	−24.0 ± 5.9
Inferolateral	22.1 ± 15.0	33.0 ± 12.2	35.4 ± 15.9	−18.0 ± 3.8	−15.4 ± 3.5	−20.2 ± 4.1
Anterolateral	29.7 ± 17.1	32.8 ± 9.9		−21.1 ± 5.2	−18.2 ± 4.9

**Table 7:** Longitudinal strain values obtained for the normal group

Segment	Horizontal long-axis			Vertical long-axis		
	Septal	Lateral	Apex	Inferior	Anterior	Apex
Basal	−27.6 ± 5.4	−25.9 ± 8.6	−14.3 ± 4.6	−20.0 ± 4.4	−19.0 ± 7.4	−14.1 ± 6.3
Mid	−16.1 ± 7.7	−9.6 ± 6.4		−18.6 ± 7.0	−20.2 ± 8.3	
Apical	−18.0 ± 8.8	−11.5 ± 4.3		−12.8 ± 6.7	−12.2 ± 5.0	

**Fig. 4:**
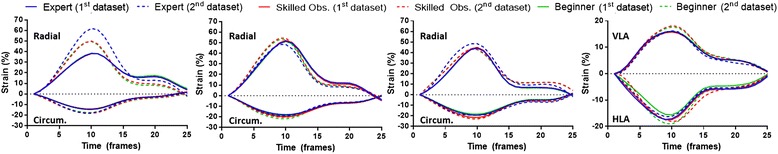
Strain curve examples from different subjects in the patient group. The continuous line represents the first acquisition, while the dashed line represent the second acquisition. Blue shows the result obtained by the Expert observer, red the result obtained with skilled observer and green the result achieved by the beginner. In order to ease the visualization, the absolute value of the longitudinal strain along the vertical long axis is used

**Fig. 5:**
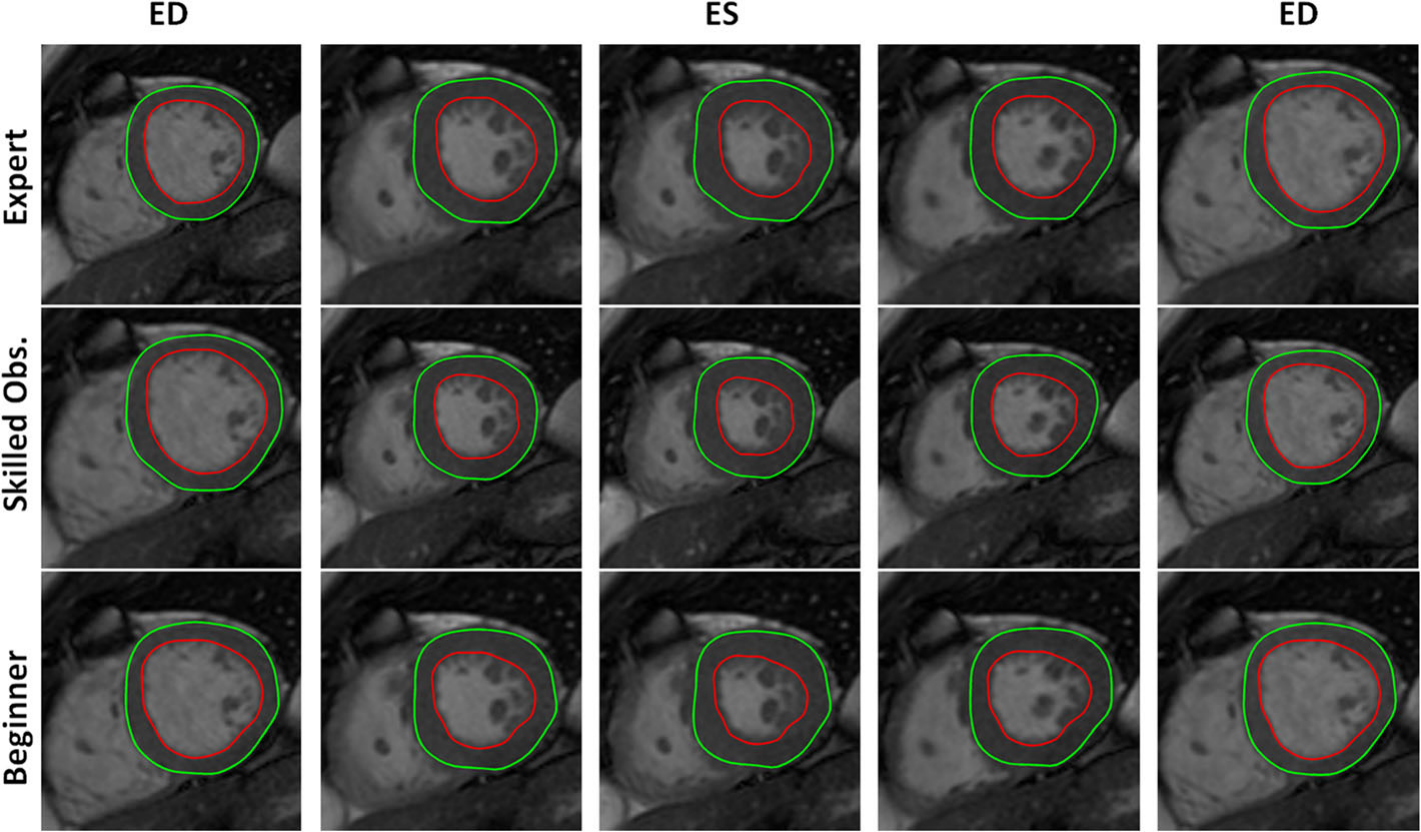
Feature tracking in cardiac short-axis in a subject belonging to the normal group by an expert, a skilled observer, and a beginner. Five time points over the cardiac cycle are shown

**Fig. 6:**
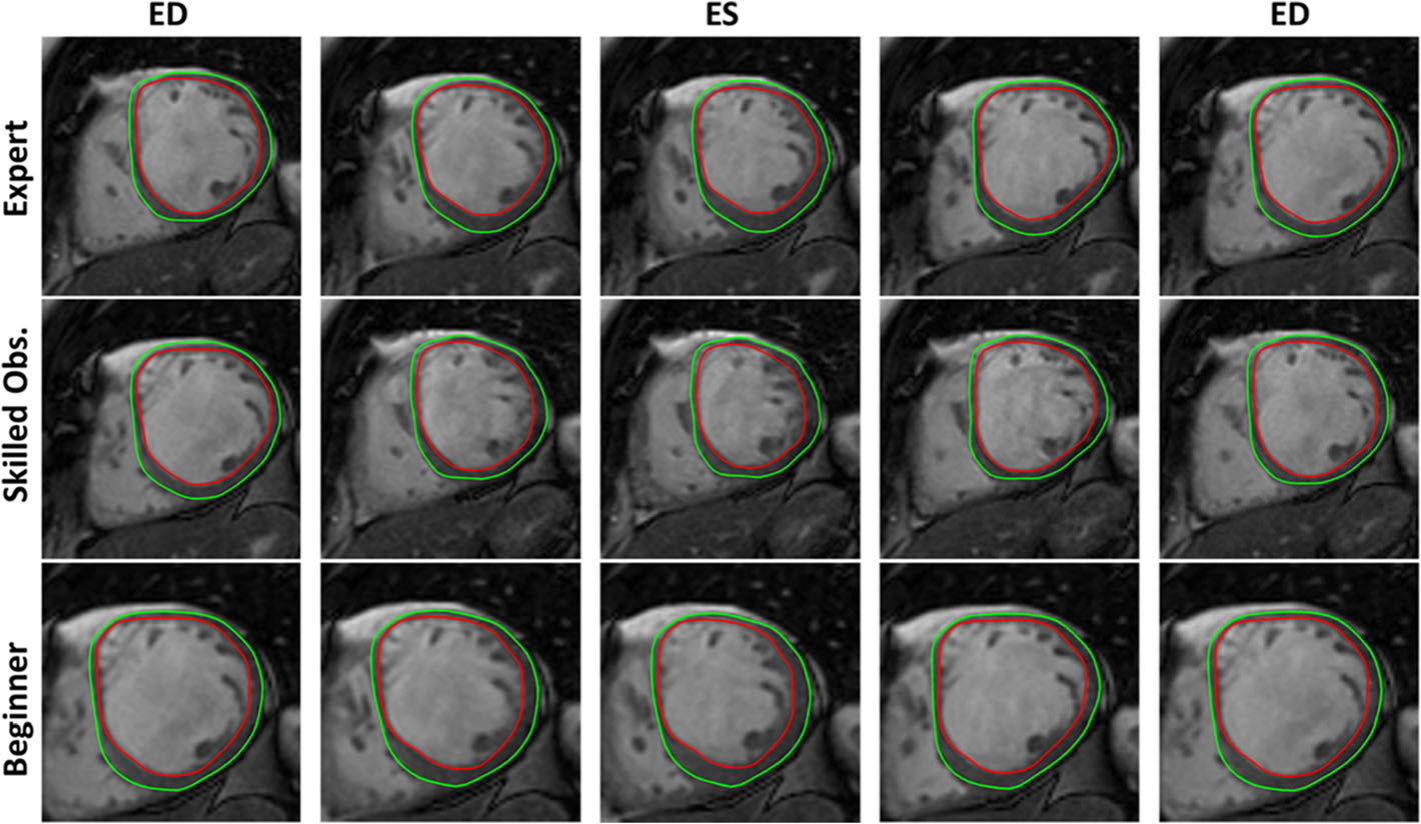
Feature tracking in cardiac short-axis in a patient with dilated cardiomyopathy by an expert, a skilled observer, and a beginner. Five time points over the cardiac cycle are shown

Inter-study variability contributed the most to the overall variability. Moreover, for inter-study variability a small, but significant bias was found in all groups for radial strain (i.e.,−2.25% to−2.53%) independent of the level of expertise of the observer (Table 5 and Fig. 2). In contrast, intra-observer variability as shown in Table 2 was low. The impact of the level of expertise on variability is shown in Tables 3, 4 and 5. Remarkably, the inter-observer variability between a skilled reader and an expert was not substantially different from the variability between a beginner and an expert (Table 3). A similar pattern was found with regard to differences in inter-study variability between observers in the healthy volunteer and patient group (Tables 4 and 5). This is also reflected in Fig. 4, showing that the differences in strain-time curves were primarily caused by test-retest variability and not by the level of expertise of the observer. As can be appreciated in Figs. 5 and 6, showing a tracking result in a normal subject and a patient with dilated cardiomyopathy, the level of experience has a limited impact of tracking of endo- and epicardial contours over the cardiac cycle.

**Fig. 7:**
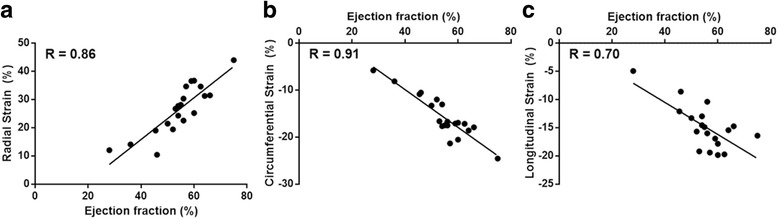
Correlation between myocardial strain (patient and normal group) and LV ejection fraction. **a**) radial strain, **b**) circumferential strain, **c**) longitudinal strain

Compared to a selected group of patients showing no obvious findings at CMR, the variability, in a group of patients presenting both focal and diffuse myocardial pathology, yielded comparable intra- and inter-observer values, e.g., intra-observer CR for global myocardial strain ranging 1.7% to 2.6% in the normal group, versus 1.6% to 2.7% in the patient group. Finally, as shown in Fig. 7, a moderate correlation was found between global myocardial strain and LV EF ranging from R = 0.70 for longitudinal strain, R = 0.86 for radial strain, to R = 0.91 for circumferential strain.

## Discussion

In the present study, we tested the variability of a novel CMR-FT software package for myocardial strain calculation using a non-rigid, elastic image registration algorithm in a group of subjects similar to a real-life clinical setting. Moreover, we tested the influence of the level of CMR expertise on study reproducibility. Overall, intra- and inter-observer and inter-study variability in terms of LOA, CV, CR and ICC yielded modest to excellent results, comparable or even superior to values reported in literature using optical flow-based CMR-FT software [16–22]. Our study results emphasize CMR-FT is a reproducible technique to study global myocardial strain and can be applied in patients with a wide variety of acute and chronic, ischemic and non-ischemic myocardial disease without significant increase in measurement variability. Moreover, the novel CMR-FT algorithm is not influenced by the level of experience, thus applicable by CMR personnel with a basic training in CMR. Time for manual contouring endo- and epicardial contours (at end-diastole) is brief and as the contours are reliably propagated over the remainder of the cardiac cycle, strain versus time curves can be acquired allowing to appreciate myocardial strain patterns during systole and diastole throughout the left ventricle. Currently, strain computation time is still considerable (13 ± 2 min). However, as strain computation is performed in the background, it does not interfere with image contouring. Moreover, further shortening of analysis time with new software releases would facilitate integration of CMR-FT in daily clinical practice.

Cine SSFP CMR has become the standard for quantification of ventricular volumes and myocardial mass [4]. Although the superior contrast between blood and surrounding myocardium with SSFP enables excellent anatomic depiction of the cardiac chambers, SSFP-based myocardial strain analysis in a routine setting has been hampered by time-consuming manual delineation of cardiac contours over the cardiac cycle and the lack of reliable (semi)-automated analysis packages. The advent of CMR-FT software opened the door towards a more routine use of LV strain analysis [11–13]. Currently, the approaches for myocardial motion and deformation estimation are threefold: optical flow-based formulation, non-rigid elastic image registration and model-based [14, 15]. Whereas TomTec (Unterschleissheim, Germany) and Circle (cvi, Calgary, Canada) software use an optical flow-based algorithm, we used a new non-rigid, elastic image registration algorithm employing image warping techniques to estimate LV cardiac motion between subsequent frames. The myocardial deformation field is parametrized using B-splines, but additional conditions are imposed to regularize the obtained deformation field in order to avoid warping solutions that are not desired or physiologically not possible. Although this algorithm was originally developed for three-dimensional myocardial deformation analysis from cardiac ultrasound, this algorithm can be applied on CMR images as well [25, 26].

Although the number of scientific papers using CMR-FT for myocardial strain analysis in normal and patients is rapidly growing, only a few papers by a relatively limited number of research groups have assessed the accuracy and variability of SSFP-based FT analysis comparing this technique to other validated CMR techniques such as SPAMM-tagging and DENSE, or to speckle-tracking echocardiography [16–22, 26, 27]. In brief, with regard to variability, these studies have shown that variability of CMR-FT equals or is superior to the other CMR and non-CMR approaches, is not influenced by magnetic field strength nor by diurnal period, while intravenous administration of gadolinium-based contrast agents significantly affects variability [19, 21, 22, 27–31]. Invariably, all studies so far have reported a substantial increase in variability at segmental compared to global level, with the lowest variability in the mid segments in short-axis direction [19, 22, 32]. Unfortunately, only a few studies so far has addressed the issue of test-retest variability as well [16, 21, 32]. Compared to reported values in literature, similar or better values for CV and ICC, and smaller LOA were found for intra-observer, inter-observer and inter-study variability in both the normal and patient group in the present study. In particular, variability for radial strain estimation was superior to optical flow-based estimates. For example, intra-observer and inter-observer coefficient of variation for global radial strain in the patient group was 4.1% and 8.6%, respectively versus 8.9% to 20.9% and 12.8% to 19.6% respectively for optical-flow based estimates [20, 33]. Intraclass correlation coefficients were 0.99 and 0.97 versus 0.75 to 0.92 and 0.55 to 0.96, respectively [20, 33]. These findings suggest that a non-rigid registration of the myocardial deformation may be more appropriate for depicting changes in radial direction than the current optical flow-based methods. Indeed, the superior performance found for the radial strain can be explained by the high number of myocardial image samples used during the non-rigid method (i.e. entire image content) and the regularization strategies applied.

Another remarkable finding in the present study is that variability does not significantly increase when applied to a real patient group showing a variety of focal and diffuse myocardial pathology. Moreover, the level of expertise does not have a major impact of myocardial strain variability. Previous work by Schuster et al. has shown that observer dependence is reduced when using CMR-FT rather than visual analysis for interpreting low-dose dobutamine stress CMR in patients with ischemic cardiomyopathy [34]. Although there was a clear difference in CMR experience (two versus seven years of experience) in their study, we opted to include an observer with a basic knowledge in cardiac imaging, opposed to a skilled and an expert observer. Our data show that CMR-FT analyses can be reliably performed by people with a basic training in cardiac imaging (basic knowledge of cardiac anatomy, cardiac imaging planes, and knowledge about the peculiarities of cardiac contouring).

Whereas global strain variability was good to excellent, segmental strain variability was moderate, questioning it use to reliably depict small changes in regional myocardial strain. For example CR for segmental radial strain in the patient groups was as high as 29.1% (Table 1). At this point, the non-rigid image registration algorithm is not superior to the optical-based approach [19, 22, 32]. Apparently, all current FT software encounter problems to reproducibly track in particular the basal and apical short-axis slices. Moreover, as our software necessitates a visual definition of the mid-part of the interventricular septum to define the 16-segment model, and not the insertion points of the right ventricle as anatomic landmarks as done by other vendors, small differences in position between readings, readers or studies may negatively impact segmental strain variability.

A systematic bias in radial strain for inter-study variability in the patient group was observed. As the second CMR study was repeated with a delay of 30 min between studies, wash-out of contrast may have interfered with delineation of the endocardial contour and thus with study variability as recently reported by Kuetting et al. [31].

Although in this study we used CMR-FT for left ventricular strain analysis, the current approach can be also applied for all cardiac chambers including the atria. Since the non-rigid method estimates the motion field between consecutive frames for all image positions instead of specific locations (e.g. myocardial boundaries), no modification is required in the current framework to evaluate strain in the remaining chambers. An example of CMR-FT of the right ventricle and left atrium is shown as additional movie video (see Additional files 2, 3 and 4).



**Additional file 2:** A movie showing feature tracking applied to the right ventricle (short-axis SSFP cine CMR) in a healthy volunteer. (AVI 1231 kb)




**Additional file 3:** A movie showing feature tracking applied to the right ventricle (horizontal long-axis SSFP cine CMR) in a healthy volunteer. (AVI 2382 kb)


### Limitations

Several limitations need to be mentioned. The aim of the present study was to report intra-observer, inter-observer and inter-study variability of a novel algorithm for CMR-FT in a group of subjects representing a real-life situation. Awaiting a head-to-head comparison between this algorithm and optical flow-based algorithms, we provided for comparison of our data some variability values as reported in literature. However, as the study groups and approaches may differ, any comparison should be taken with the necessary caution. Moreover, at least two groups have recently published age- and gender related values for CMR-FT based myocardial strain [30, 31]. The current values from the ‘normal’ group cannot be considered as reference values because all subjects had complaints and were therefore referred to CMR. Nevertheless, these strain values compare rather well with the “normal” healthy volunteers as reported by Schuster et al. [19].

## Conclusions

We tested a non-rigid, elastic image registration algorithm for CMR-FT-based myocardial strain analysis in a real-life clinical setting. Variability values seem equally or superior than those reported in literature with optical flow-based approaches. Moreover, strain analysis can be reproducibly performed by observers with a different level of CMR expertise in patients presenting a wide range of myocardial diseases. Further improvement, however, is needed to reliably depict subtle changes in segmental strains.

## Additional files

Additional file 1: Shows the demographics of the healthy volunteer, and the subjects in the normal and patient group. (DOCX 17 kb)
Additional file 4: A movie showing feature tracking applied to the left atrium (horizontal long-axis SSFP cine CMR) in a healthy volunteer. (AVI 2111 kb)

## Abbreviations

CMR: Cardiovascular magnetic resonance
CMR-FT: Cardiovascular magnetic resonance – feature tracking
CR: Coefficient of repeatability
CV: Coefficient of variation
Ecc_SAX_: Circumferential strain in short-axis
Ell_LAX_: Longitudinal strain in long-axis
Err_SAX_: Radial strain in short-axis
FT: Feature tracking
ICC: Intraclass correlation coefficient
LOA: Limits of agreement
LV: Left ventricle
SSFP: Steady-state free-precession
