# Supplementary material for: Cardiovascular magnetic resonance myocardial feature tracking using a non-rigid, elastic image registration algorithm: assessment of variability in a real-life clinical setting
Source: J Cardiovasc Magn Reson. 2017 Feb 17;19:24. doi: 10.1186/s12968-017-0333-y (PMC5314711; doi:10.1186/s12968-017-0333-y)
Supplement: Additional file 1: — Shows the demographics of the healthy volunteer, and the subjects in the normal and patient group. (DOCX 17 kb) [file 12968_2017_333_MOESM1_ESM.docx]

Additional File 1

**Heart rate, time of end-systole and LV volumes, mass and ejection fraction in the healthy volunteer**

|  | **Heart rate (bpm)** | **Time ES (ms)** | **EDV (ml)** | **ESV (ml)** | **SV (ml)** | **EF(%)** | **Mass (g)** |
| --- | --- | --- | --- | --- | --- | --- | --- |
| 1 | 66 | 309 | 192 | 68 | 124 | 64,6 | 138 |
| 2 | 61 | 297 | 191 | 62 | 129 | 67,6 | 130 |
| 3 | 57 | 316 | 193 | 64 | 129 | 66,8 | 139 |
| 4 | 60 | 302 | 196 | 68 | 128 | 65,2 | 129 |
| 5 | 59 | 303 | 195 | 68 | 126 | 64,9 | 141 |
| 6 | 60 | 304 | 196 | 67 | 129 | 66,0 | 139 |
| 7 | 63 | 309 | 192 | 65 | 126 | 65,9 | 135 |
| 8 | 60 | 304 | 197 | 60 | 137 | 69,3 | 137 |
| 9 | 59 | 308 | 191 | 61 | 131 | 68,4 | 132 |
| 10 | 64 | 302 | 189 | 58 | 131 | 69,3 | 133 |
| Mean(STD) | 61(3) | 305(5) | 193(3) | 64(4) | 129(2) | 66.8(1.8) | 135(4) |

ES – End-systole; EDV – end-diastolic volume; ESV – end-systolic volume; SV – Stroke Volume; EF – Ejection fraction;

**Normal group**

| Subject | **Clinical referral** | **HR** | **EDV** | **EDV** | **ESV** | **SV** | **EF** | **Mass** |
| --- | --- | --- | --- | --- | --- | --- | --- | --- |
| 1 | ventricular arrhythmias | 77 | 166 | 166 | 60 | 106 | 64 | 134 |
| 2 | non-sustained ventricular tachycardia | 46 | 158 | 158 | 64 | 94 | 60 | 75 |
| 3 | ventricular extrasystoles | 62 | 206 | 206 | 94 | 112 | 55 | 104 |
| 4 | palpitations, ventricular extrasystoles right ventricular outflow tract | 66 | 187 | 187 | 88 | 99 | 53 | 93 |
| 5 | familial history of dilated cardiomyopathy, syncope | 67 | 160 | 160 | 60 | 101 | 62 | 99 |
| 6 | recurrent atrial fibrillation | 63 | 204 | 204 | 94 | 110 | 54 | 113 |
| 7 | centricular extrasystoles | 84 | 142 | 142 | 61 | 81 | 57 | 64 |
| 8 | myocardial hypertrophy | 62 | 187 | 187 | 94 | 94 | 50 | 105 |
| 9 | sarcoidosis | 57 | 233 | 233 | 121 | 112 | 52 | 124 |
| 10 | marathon runner with abnormal ECG | 59 | 164 | 164 | 57 | 107 | 66 | 124 |

EDV – end-diastolic volume; ESV – end-systolic volume; HR – heart rate (beats per min); SV – Stroke Volume; EF – Ejection fraction;

**Patient group**

| Subject | **clinical referral / diagnosis** | **HR** | **EDV** | **ESV** | **SV** | **EF** | **Mass** |
| --- | --- | --- | --- | --- | --- | --- | --- |
| 1 | hypertrophic cardiomyopathy | 53 | 118 | 29 | 88 | 75 | 83 |
| 2 | mild dilated cardiomyopathy | 63 | 154 | 87 | 67 | 45,5 | 81 |
| 3* | ventricular extrasystoles | 53 | 155 | 69 | 86 | 56 | 97 |
| 4 | acute myocardial infarction | 70 | 112 | 60 | 52 | 46 | 114 |
| 5 | cardiac amyloidosis | 58 | 135 | 58 | 76 | 56 | 119 |
| 6 | acute myocarditis | 69 | 196 | 91 | 105 | 54 | 117 |
| 7 | old inferior myocardial infarction | 52 | 182 | 72 | 110 | 60 | 107 |
| 8* | familial history of hypertrophic cardiomyopathy | 86 | 125 | 52 | 74 | 59 | 108 |
| 9 | ischemic cardiomyopathy | 55 | 233 | 169 | 64 | 28 | 80 |
| 10 | acute myocardial infarction | 55 | 197 | 126 | 72 | 36 | 195 |

EDV – end-diastolic volume; ESV – end-systolic volume; HR – heart rate (beats per min); SV – Stroke Volume; EF – Ejection fraction;

* normal CMR findings in patient 3 and 8
